# An observational longitudinal study of the use of ROX index to predict treatment failure in patients receiving continuous positive airway pressure for COVID‐19

**DOI:** 10.1002/hsr2.482

**Published:** 2022-01-12

**Authors:** Asfandyar Yousuf, David Shimon Gottlieb, Aneesh Aggarwal, Bernadette Peacock, Shruthi Konda

**Affiliations:** ^1^ Respiratory medicine Watford General Hospital Watford UK

## 1 INTRODUCTION

Five percent of patients with COVID‐19 develop the critical disease, with one of the complications being acute hypoxemic respiratory failure (AHRF) requiring advanced respiratory support.^1^ In April 2020, the British Thoracic Society (BTS) issued guidance for the delivery of continuous positive airway pressure (CPAP) to patients with AHRF as a bridging therapy prior to escalation to the intensive care unit (ICU).^2^ This therapy has been shown to benefit patients with AHRF due to COVID‐19 in a large multi‐center trial RECOVERY‐RS, their findings are still awaiting peer review.^3^ Evidence is needed on how to safely manage these patients outside of ICU and prevent unnecessary delay of intubation.^4^

The use of ROX index (ratio of pulse oximetry/fraction of inspired oxygen to respiratory rate) was validated in 2019 to predict whether patients with AHRF can be safely managed on HFNO or if invasive mechanical ventilation (IMV) should be considered.^5^ ROX index has also been studied in patients with COVID‐19 on HFNO and higher values were found to be associated with HFNO success.^6^, ^7^

This study investigated whether there is an association between ROX index before starting CPAP and within the first 24 hours of starting it and our outcome measure ‐ whether patients were weaned off or failed CPAP. Failure was defined as either death or needing IMV depending on their treatment escalation plan.

## 2 METHODS

This was an observational longitudinal study of patients admitted to the acute respiratory unit dedicated to delivering CPAP at Watford General Hospital, UK, from March 15th to April 28th, 2020, for COVID‐19 related AHRF. Inclusion criteria were adult patients (18 years and above) with AHRF with either positive PCR test for SARSnCOV2 or clinical picture was consistent with COVID‐19.

Data collection was part of routine service evaluation with prior departmental approval, data analysis was retrospective and did not affect patient outcomes. Research ethical approval was therefore not required. Medical notes and observation charts were used to review clinical events and calculate ROX index values.

CPAP delivery was in line with BTS guidance.^2^ CPAP was commenced at a PEEP of 5 and then PEEP and FiO2 were gradually up titrated guided by peripheral saturations. The intensive care team would become involved with patients who were deemed suitable for full escalation, deteriorating on CPAP and would potentially require invasive mechanical ventilation. Some of the patients stayed on CPAP because of their agreed treatment escalation plan, while those appropriate for intubation, either stayed on the ward or were taken to ICU for IMV depending on clinical course.

ROX index was calculated on admission, before patients were started on CPAP, and at 2, 6, 12, 18, and 24 hours (hr) after CPAP was started. For each category, the patients in each were split according to whether they survived or died. The mean values in each category, the SD and the count were isolated. These were used to calculate the One‐Tailed Z‐statistic ‐ the proportion of the normal distributions overlapping, which were converted into p values.

Microsoft Excel 2010 and IBM SPSS Statistics Release 27.0.0.0 were used for data collection and analysis.

The primary outcome was whether patients were weaned off or failed CPAP.

## 3 RESULTS

Medical notes for 101 patients admitted for CPAP were reviewed. Two patients were excluded as other diagnoses were possible. Medical notes were unavailable for 4 patients at the time of data collection, which left 95 patients for final analysis.

The mean and median age was 66.4 (SD 12.2) and 66 years (lower interquartile 57, upper interquartile 75) respectively, age range was 40‐90. There was a male preponderance at a 2:1 ratio (65 males:30 females *P* = .0072, One‐Tailed Fisher's Exact Test). The likelihood of a successful wean based on gender did not pass the test of significance (*P* = .81, Two‐Tailed Fisher's Exact Test). Sixty eight out of 95 patients failed CPAP (71.6% failure rate). The failure rate in those for full escalation was 55.6% (30/54) and 92.7% (38/41) in those not for intubation.

**FIGURE 1:**
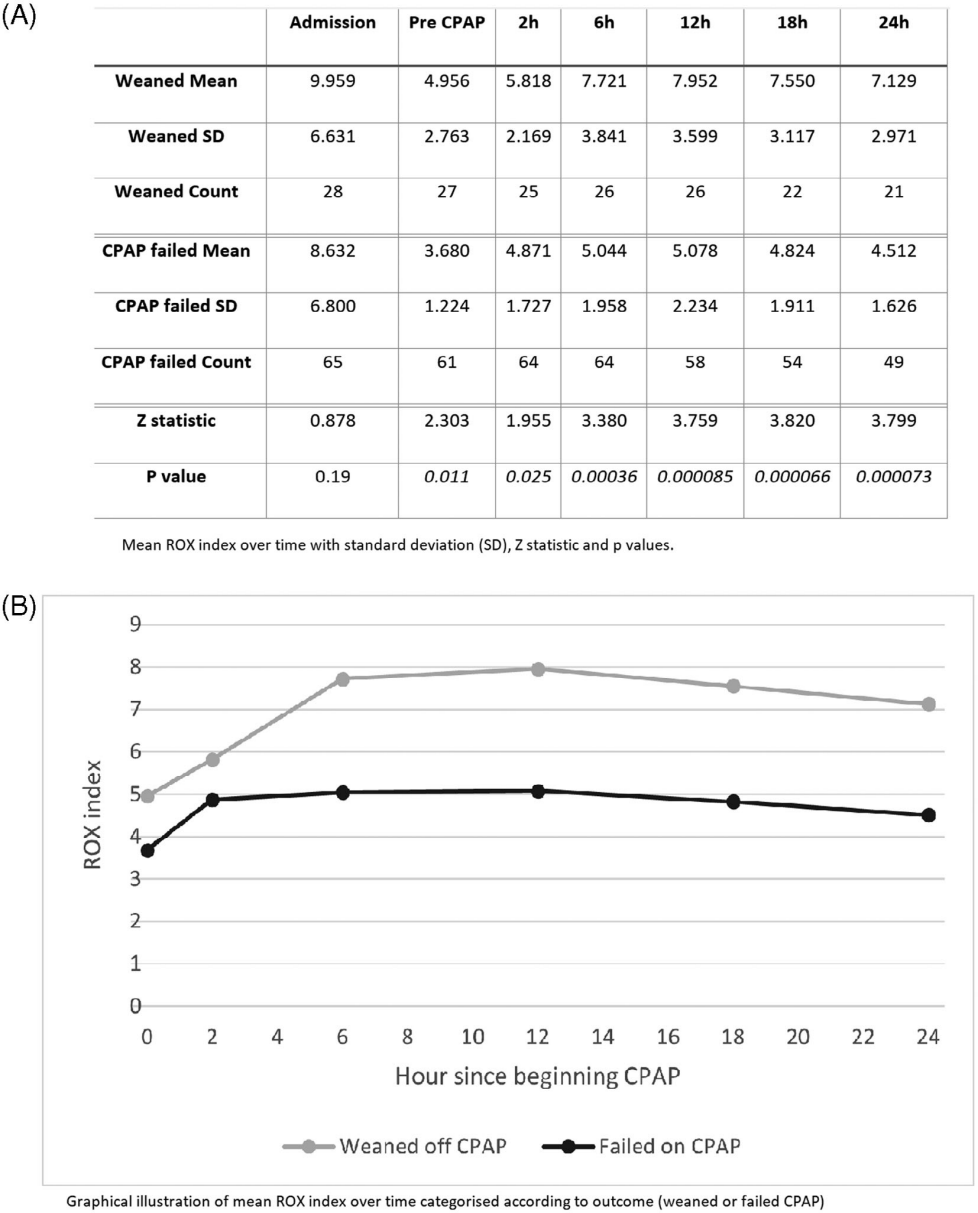
Mean ROX index over time categorised according to outcome (weaned or failed CPAP)

ROX index was not available for every patient at every time point, values were used where available. The mean values in each category, the SD and the count were used to calculate the One‐Tailed Z‐statistic ‐ the proportion of the normal distributions overlapping, which were converted into p values (Figure 1A).

ROX index was significantly greater in the group that was weaned compared to the group that failed CPAP (Figure 1B). ROX index on admission did not meet the test of significance for those who were weaned vs those who failed CPAP.

**FIGURE 2:**
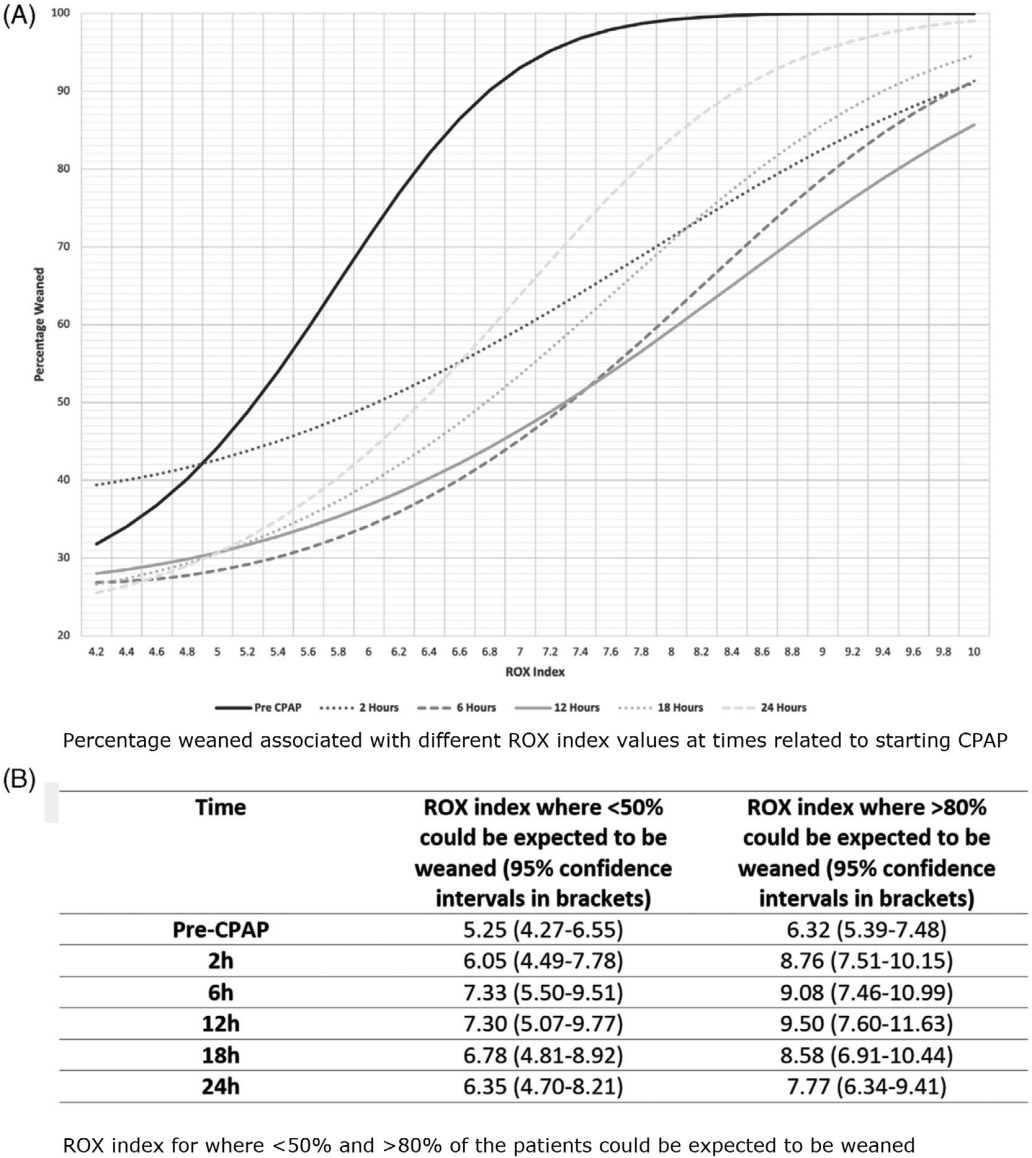
CPAP wean expectancy based on ROX index at specified timepoints

The means and standard deviations for the weaned vs failed groups were compared and the odds ratio graph was converted into a “success versus failure” graph (Figure 2A).

The ROX index in each time category were modeled in accordance with a normal distribution in each outcome category. The simultaneous equation of weaned to failed was solved at the 50:50 and 80:20 ratio, before extremes of the model were reached.

ROX index for where <50% and > 80% of the patients could be expected to be weaned are detailed in Figure 2B.

## 4 DISCUSSION

Roca et al reported that ROX index of 4.88 and above at 12 hours predicted HFNO success and ROX less than 3.85 at 12 hours predicted HFNO failure (5). This data was reported before the current pandemic and included patients who did not have COVID‐19 related AHRF. Calligaro et al recently reported the use of ROX index at 6 hours in patients with COVID 19 on HFNO and identified values of ≥3.77 as predictive of success and ≤2.2 as predictive of failure in a multi‐center prospective study.^6^ Zucman N et al reported the use of ROX index calculated within the first 4 hours of initiating HFNO as a useful measure to identify patients who are likely to fail on HFNO.^7^

These studies looked at patients who were already in ICU and were receiving HFNO for respiratory support. However, we looked at the utility of the ROX index as a prognostic indicator in patients receiving CPAP on a respiratory ward. Our results show that ROX index before starting CPAP and within 24 hours of initiation can differentiate patients with COVID‐19 related AHRF who are likely to wean or fail CPAP. Increasing significance of ROX index' association with time could be suggestive of increasing prognostic potential of ROX index with time. This could allow ROX index as a monitoring tool for patients being managed outside of ICU, pending validation in stronger studies. By definitions used above, a pre‐CPAP ROX>6.32 is indicative of a successful wean in >80% cases. In the appropriate clinical context, a lower ROX index which is trending down with time would be indicative of consideration of intubation in the full escalation group and in the non‐escalation group could aid prognostication. Though this data was retrospective and the patient population was unselected, the significance of these findings warrants further research with larger prospective studies.

## FUNDING

No funding sources to declare.

## CONFLICT OF INTEREST

The authors have no conflict of interest to declare.

## AUTHOR CONTRIBUTIONS

Conceptualization: Asfandyar Yousuf, Shruthi Konda.

Data acquisition: David Gottlieb, Bernadette Peacock, Asfandyar Yousuf.

Formal Analysis: Aneesh Aggarwal.

Writing—Review and Editing: Asfandyar Yousuf, David Gottlieb, Aneesh Aggarwal, Bernadette Peacock, Shruthi Konda.

Writing—Original Draft: Asfandyar Yousuf, David Gottlieb, Aneesh Aggarwal.

All authors have read and approved the final version of the manuscript [Asfandyar Yousuf] had full access to all the data in this study and takes complete responsibility for the integrity of the data and the accuracy of the data analysis.

Asfandyar Yousuf affirms that this manuscript is an honest, accurate, and transparent account of the study being reported; that no important aspects of the study have been omitted; and that any discrepancies from the study as planned (and, if relevant, registered) have been explained.

## Data Availability

Data available on request from the authors.
